# LncRNA LOC285194 modulates gastric carcinoma progression through activating Wnt/β‐catenin signaling pathway

**DOI:** 10.1002/cam4.2844

**Published:** 2020-01-28

**Authors:** Bingzheng Zhong, Qiang Wang, Jiali He, Yi Xiong, Jie Cao

**Affiliations:** ^1^ Department of General Surgery, Guangzhou Digestive Disease Center Guangzhou First People's Hospital School of Medicine South China University of Technology Guangzhou China

**Keywords:** apoptosis, Gastric cancer (GC), invasion, LOC285194, long noncoding RNAs (lncRNAs), migration, proliferation

## Abstract

Emerging evidences have revealed long noncoding RNAs (lncRNAs’) critical roles in diverse human carcinoma. Among these cancers, lncRNA LOC285194 has been extensively investigated in several types of carcinomas in the recent years. Nevertheless, the biological function, clinical relevance, and the influence of LOC285194 in gastric cancer (GC) are not fully understood. The present study aims to explore the biological function of LOC285194 in the progression and development of GC. First, LOC285194 expressions were detected in GC tissues and cell lines. The functional role of LOC285194 in GC was evaluated both in vitro and in vivo. Our data found that LOC285194 was lowly expressed both in human GC tissues and GC cell lines compared with corresponding normal controls. Moreover, LOC285194 was mitigated by transfection with LV‐LOC285194 in both HGC‐27 and MKN45 cell lines. Silencing of LOC285194 remarkably induced GC cell livability and cell proliferation. On the contrary, the LOC285194 overexpression suppressed MKN45 and HGC‐27 cell proliferation and promoted cell apoptosis. Additionally, silencing of LOC285194 increased the ability of colony formation, cell migration, and invasive capacities, together with blocking the apoptotic rates of GC cells. Correspondently, LOC285194 overexpression exerted the opposite effects. Mechanistically, silencing of LOC285194 promoted GC progression via inducing Wnt signaling activity. Moreover, in vivo xenografts nude mice model results showed that LOC285194 inhibited GC progression through targeting Wnt signaling. Taken together, LOC285194 is associated with GC progression by regulating the Wnt signaling transduction, potentiating LOC285194's promising role as a novel treatment biomarker in GC.

## Graphical abstract

LOC285194 inhibited GC progression through targeting Wnt signaling. Taken together, LOC285194 is associated with GC progression by regulating the Wnt signaling transduction, suggesting that LOC285194 might serve as a novel treatment target in GC.

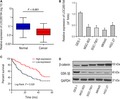

## 1 INTRODUCTION

Gastric cancer (GC) ranks as the second leading cause of cancer‐induced fatality, which belongs to gastrointestinal malignant tumors—most commonly seen worldwide.^1^, ^2^ For most patients with GC, GC was diagnosed to have already being progressed into advanced tumor stages, which are associated with malignant proliferation, extensive invasion as well as lymphatic metastasis.^3^ To date, limited clinical therapeutic options with successful outcomes are available for this disease, which brings about relatively high mortality rate.^4^ Long noncoding RNAs (lncRNAs) are noncoding RNAs with more than 200 nucleotides, which have no capability to encode proteins.^5^ Multiple investigations demonstrated that lncRNA plays substantial roles in cell development, differentiation as well as other biological process.^6^ Mechanistically, lncRNA acts as a ceRNA to sponge target miRNA response elements and imposing its post‐transcriptional regulatory level. For instance, DANCR is highly expressed in late stage of cancers and is correlated with advanced cancer progression and poor OS of colorectal cancer.^7^ LncRNA UCA1 promotes the chemical drug resistance of bladder carcinoma through modulating Wnt signaling pathway.^8^ Additionally, Linc00152 is involved in cell apoptosis, cell cycle arrest, epithelial‐mesenchymal transition (EMT), as well as cell invasion and migration of gastric cancer.^9^ HOX HOTAIR is dysregulated and is related to GC progression. Besides, maternal expression 3 (MEG3) is attenuated in GC, which inhibits GC development via regulating miRNA‐21 (miR‐21).^10^ Moreover, PVT1 promotes GC progression by upregulating FOXM1.^11^ Nevertheless, the significance of LOC285194 in GC is still unclear.

In this study, we investigated the expression of LOC285194 and downstream Wnt pathway in GC cells. We also performed gain‐ and loss‐of function studies of LOC285194 to elucidate its role in GC progression. Our findings indicated that Wnt signaling transduction was notably inhibited by elevated LOC285194. Taken together, our investigation revealed that LOC285194 downregulation facilitates GC progression via activating Wnt signaling pathway.

## 2 MATERIALS AND METHODS

### 2.1 Study cohorts

A cohort of 72 paired GC tissues and adjacent noncarcinoma control tissues were obtained from patients who received surgery at Guangzhou First People's Hospital between 2014 and 2015. Patients who accepted local or systemic treatment before surgery were excluded. The clinical characteristics of GC patients are listed in Table S1. This study was approved by the research ethics committee of Guangzhou First People's Hospital (Approval number: GFPH‐20140125).

### 2.2 Cell culture

MGC‐803, HGC‐27, AGS, MKN45, and GES‐1 cells were obtained from the Cell Bank of Chinese Academy of Sciences (Shanghai), which were cultured in Dulbecco modified Eagle medium (DMEM) (Invitrogen) containing penicillin (100 IU/mL), and streptomycin (0.1 mg/mL, HyClone) in a humidified incubator containing 5% CO_2_ at 37°C.

### 2.3 Lentivirus vector infection

LOC285194 (LV‐LOC285194 group), lentivirus negative control (LV‐NC group) and LV‐shRNA group were purchased from Santa Cruz Biotechnology, Inc We synthesized and subcloned the LOC285194 sequence into plasmid pCDH‐CMV‐MCS‐EF1‐coGFP and then transferred with lentivector, to produce pCDH‐CMV‐MALAT1‐EF1‐coGFP (GenePharma, Shanghai, China). Prior to the transfection, cells (2 × 10^5^) were seeded into 6‐well plates (Corning, America) and cultured overnight. The transfection was performed with the aid of Lipofectamine 2000 reagent (Invitrogen) in accordance with the manufacturer's protocol.

### 2.4 Cell counting kit‐8 detection

A total of 2 × 10^3^ cells was plated into 96‐well plates. Ten microliters of CCK‐8 (Dojindo Molecular Technologies) solution were supplemented at 0, 24, 48, 72, and 96 hours. A microtiter plate reader was applied to measure the optical density (OD) at 450 nm. The cell survival rates were presented as the absorbance. A total of six replicates were calculated under the same conditions to represent the results.

### 2.5 EDU assay

To detect cell proliferation, 5‐acetylene‐2'‐deoxyuridine (EDU, RiboBio) was applied to perform cell proliferation test. After 48 hours after the infection, the cells were incubated with 50 mu MEdU for 2 hours. Then, EdU positive cells were determined.

### 2.6 Colony formation test

A total of 1 × 10^3^ cells was inoculated into 6‐well plates, which were displayed in the medium with 10% FBS. Then, the cells were fixed with methanol and then 0.1% crystal violet (Sigma‐Aldrich) was applied to stain the cells 2 weeks later.

### 2.7 Flow cytometry analysis

Apoptosis rate was determined with an annexin V‐FITC/PI apoptosis detection kit (Solarbio Life Sciences) using a BD LSR Fortessa flow cytometer (BD Biosciences). Next, the cells were stained with fluorescein isothiocyanate (FITC)‐serotonin V and propylene iodide (PI), which were then washed with cold PBS three times post‐transfection. Then, the cells were suspended with 1 × binding buffer. Next, the cells were re‐suspended in 1 mL 1 × binding buffer. Meanwhile, we transferred 100 μL cell suspension into another tube, followed by the adding of 5 μL annexin V‐FITC. After the cells were incubated at room temperature for 10 minutes away from light, 5 μL PI was added and then incubated for 5 minutes at room temperature in the dark. All samples were detected with a FACSCalibur flow cytometer (Beckman Coulter).

### 2.8 Transwell assay

The cells were migrated through a polycarbonate membrane with 8 μm pore‐size, and cell suspension was added to the upper compartment of Transwell, followed by matrix gel invasion detection on 100 000 cells. After incubation for 24 hours, the filter membranes in the chambers were removed, followed by washing with PBS thrice to remove the medium and Matrigel. Next, we applied cold methanol to fix the cells for 30 minutes, which were then stained with 0.1% crystal violet and counted under a microscope.

### 2.9 QRT‐PCR

For extraction of total RNA, TRIzol reagent (Invitrogen) was employed. Prime Script TM RT Master Mix was applied for RNA reverse transcription, and SYBR Premix Ex Taq II (TaKaRa Bio Technology) was adopted for qPCR, followed by ABI 7500 Real‐Time System (Life Technologies) for real‐time PCR. The cycle conditions were set as below: predenaturation for 10 minutes at 95°C; 35 cycles for 15 seconds at 95°C, 55°C for 60 seconds. The primers were as follows: 5ʹ‐TTATGGACCTCCATTCGCCC‐3ʹ, 3ʹ‐GCACGCCTATGGACTCACTT‐5ʹ. β‐actin: 5ʹ‐AAAGACCCCTACGTGAACA‐3ʹ, 3ʹ‐TGGCCTGCTTTCATACTGC‐5ʹ.

### 2.10 Western blotting

We used RIPA buffer containing a mix of protease inhibitors to lysate the total protein. Thirty micrograms of protein lysates were separated by SDS‐PAGE and then transferred onto PVDF membranes. The anti‐β‐catenin antibody (ab2365, 1:800, Abcam), the anti‐GSK‐3β antibody (1:800; Cat#9336, Cell signaling, MA) was adopted as primary antibodies. Anti‐GAPDH antibody (ab14247, 1:1000, Abcam) was used as internal references. We applied peroxidase‐conjugated GOAT anti‐Rabbit IgG‐horseradish peroxidase (1:1000; Abcam) as secondary antibodies and exposed protein bands with EZECL chemiluminescence detection kit (Millipore) for horseradish peroxidase (HRP). The acquired bands were normalized and quantified by ImageJ software.

### 2.11 Animal studies

Animal experiments were performed according to the National Institutes of Health guidelines for the use and care of animals for trial. Male nude Balb/C mice (5‐6 weeks) were fed in the animal house of Experimental Center, Guangzhou First People's Hospital. The mice were divided into LV‐NC group: mice were inoculated subcutaneously into the right flank with HGC‐27 cells (5 × 10^6^) infected with lentivirus negative control (n = 5); LV‐shRNA group: mice were inoculated with HGC‐27 cells infected with Lv‐LOC285194. The tumor volume (*V*) was calculated as follows: *V* = (*ab* ^2^)/2, “*a*” represents long diameter of the tumor, and “*b*” represents the short diameter of the tumor mass. The study was approved by the Science and Technology Institutional Animal Care and Use Committee of Guangzhou First People's Hospital, School of Medicine, South China University of Technology (S0524). All the animal experiments were repeated in triplicates, respectively.

### 2.12 Immunohistochemistry and hematoxylin‐eosin (H&E) staining

We adopted 10% formalin to fix the tumor tissues, and then we sliced them into 5 μm sections for immunohistochemical analysis. Then, we deparaffinized the paraffin‐embedded tumor sections, which were then treated with citric acid buffer (pH7.0) to retrieve the heat‐induced antigen, followed by successive incubation in 3% hydrogen peroxide and 5% normal goat serum. Next, we used the polyclonal rabbit antibody against Ki‐67 (1:500; Abcam, Cambridge, Britain) to stain the sections, and fixed tumor tissues with 10% formalin overnight. Finally, we applied hematoxylin‐eosin (HE; Beijing Solarbio Science & Technology Co., Ltd., Beijing, China) to perform the staining on the sections.

### 2.13 Statistical analysis

SPSS version 17.0 Statistics was applied to perform all statistical calculations. We conducted at least 3 independent trials for each test. Then, we analyzed the variations of the two groups with a 2‐tailed Student's *t* test. We evaluated the differences by ANOVA, Dunnett's multiple comparison post‐test among groups. The *P* < .05 was deemed as significant difference.

## 3 RESULTS

### 3.1 LOC285194 expression was impaired in GC cells and Wnt/β‐catenin signaling pathway was activated

**Figure 1:**
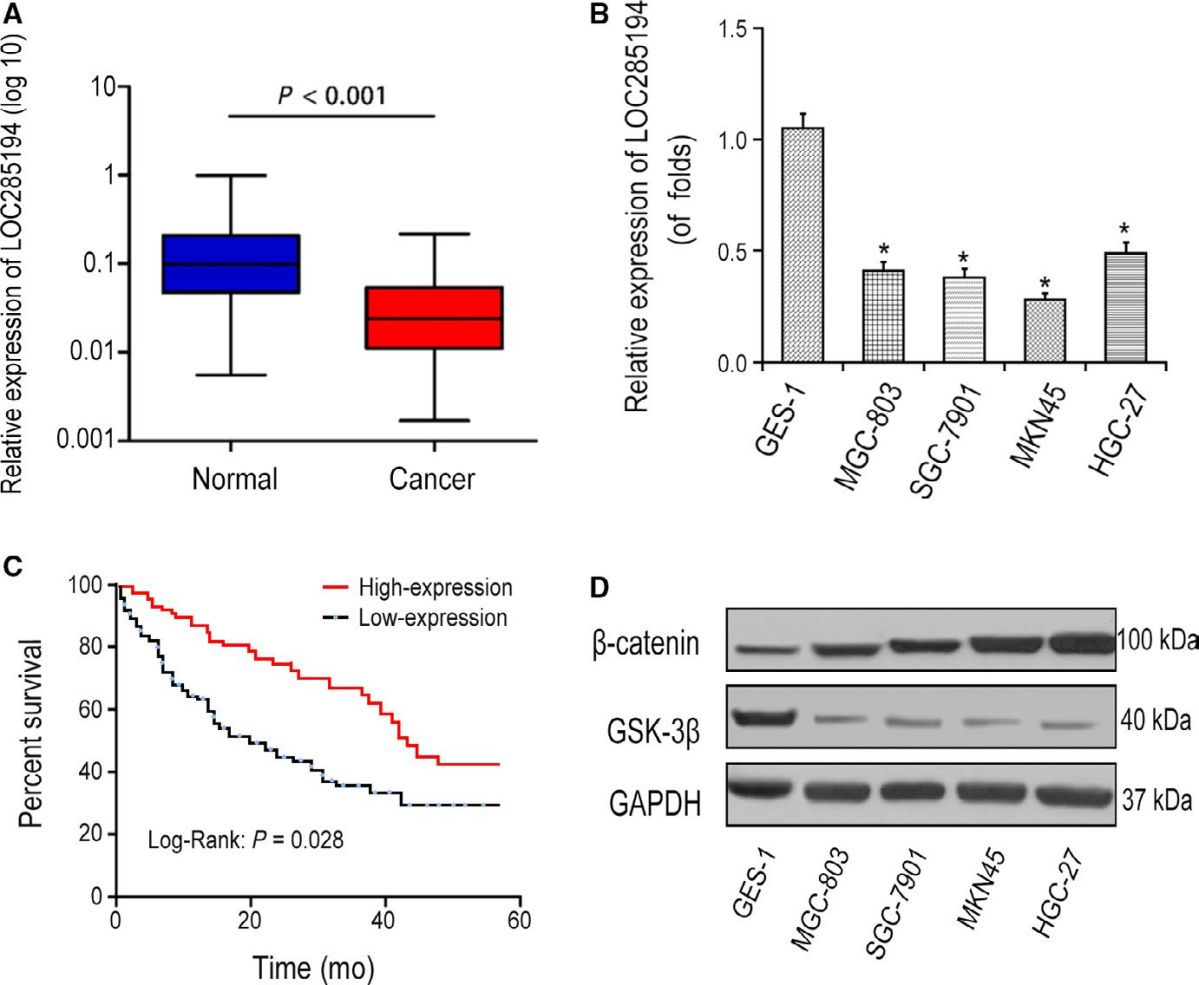
Expression of LOC285194 in GC cells. A‐B, LOC285194 expression in GC tissues and GC cell lines detected by qRT‐PCR. *P < .05. C, Kaplan‐Meier curve showed the overall survival in GC patients according to lncRNA LOC285194 expression. Red curve represents patients with high LOC285194 expression, while blue curve represents low LOC285194 expression according to the median value of LOC285194 expression. D, Protein expressions of β‐Catenin and GSK‐3β in MGC‐803, AGS, MKN45, HGC‐27, and GES‐1 cells detected by western blotting

To assess the expression levels of LOC285194, we detected LOC285194 expression in GC tissues and corresponding para‐carcinoma cells, as well as in GC cell lines, including AGS, MGC‐803, MKN45 and HGC‐27 cells and primary normal cervical squamous cells (GES‐1). As shown in Figure 1A‐B, compared with GES‐1 cells, LOC285194 expression was remarkably reduced in GC tissues and GC cells. Kaplan‐Meier analysis showed that GC patients with high lncRNA LOC285194 expression had higher overall survival rate than those with low LOC285194 expression (*P* = .028, Figure 1C). Notably, Wnt/β‐catenin signaling was remarkably triggered in GC cells (Figure 1D) compared with normal control cells. Lower LOC285194 expression levels were substantially correlated with larger tumor size (*P* = .028), higher invasion depth (*P* = .004), advanced histologic stage (*P* = .036) and lymph node metastasis (*P* = .008) in GC patients (Table S1). The findings suggested the aberrant expression of LOC285194 was correlated to GC progression.

### 3.2 LOC285194 inhibited GC cell proliferation, migration, invasion and triggered cell apoptosis

**Figure 2:**
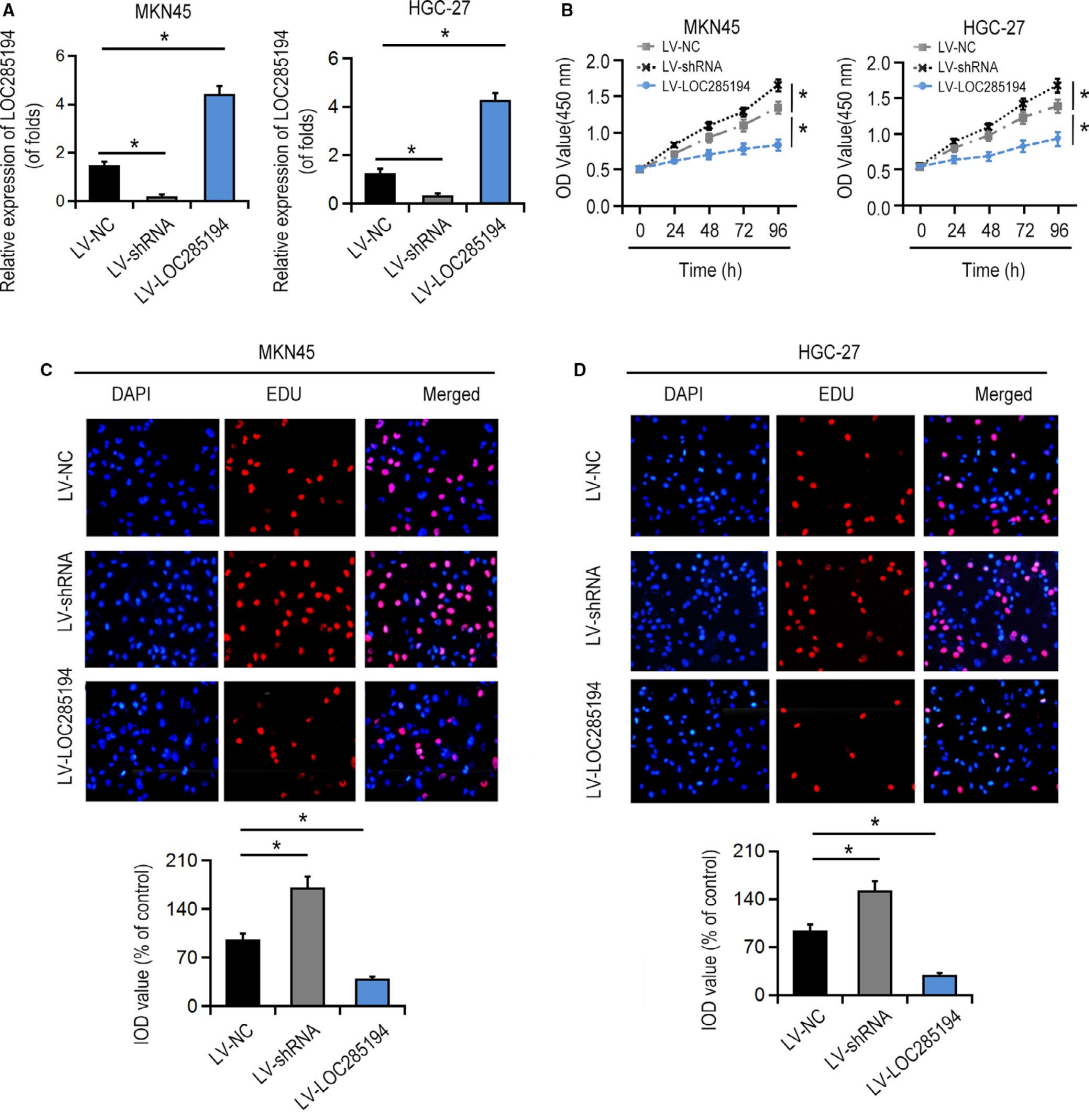
Effects of LOC285194 on GC cell proliferation. A, LOC285194 expression in MKN45 and HGC‐27 cells. Cells were infected with LV‐shRNA or LV‐LOC285194 for 48 h. B, Effects of LOC285194 on the cell proliferation of MKN45 and HGC‐27 cells detected by CCK8 assay. C‐D, Effects of LOC285194 on cell proliferation of MKN45 and HGC‐27 cells detected by EDU assay. *P < .05

**Figure 3:**
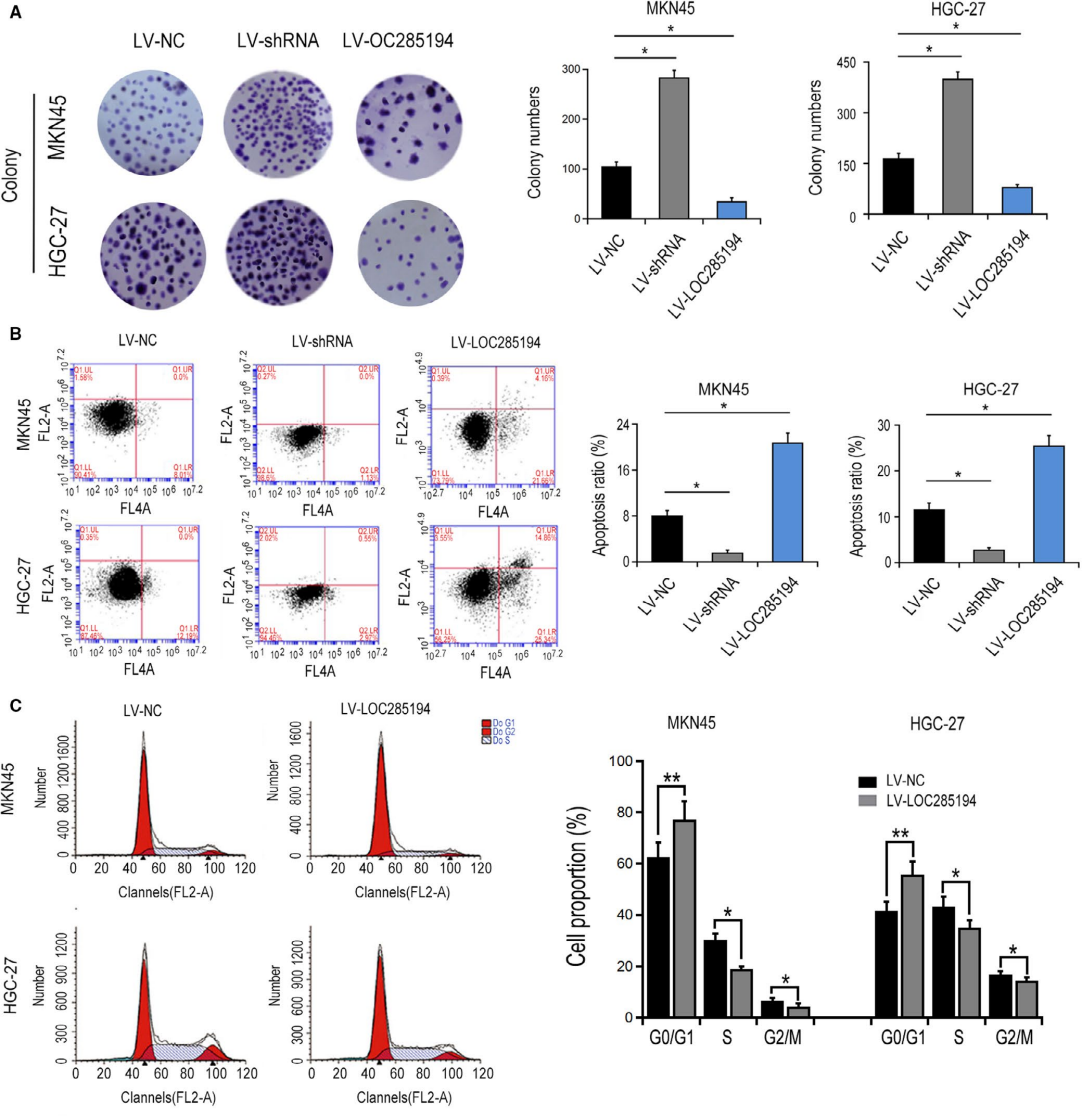
Effects of LOC285194 on GC formation ability and cell apoptosis. A, Effects of LOC285194 on the cell formation ability of MKN45 and HGC‐27 cells. B, Effects of LOC285194 on the cell apoptosis of MKN45 and HGC‐27 cells detected by flow cytometry assay. C, Effects of LOC285194 on the cell cycle of MKN45 and HGC‐27 cells detected by flow cytometry assay. *P < .05; **P < .01

**Figure 4:**
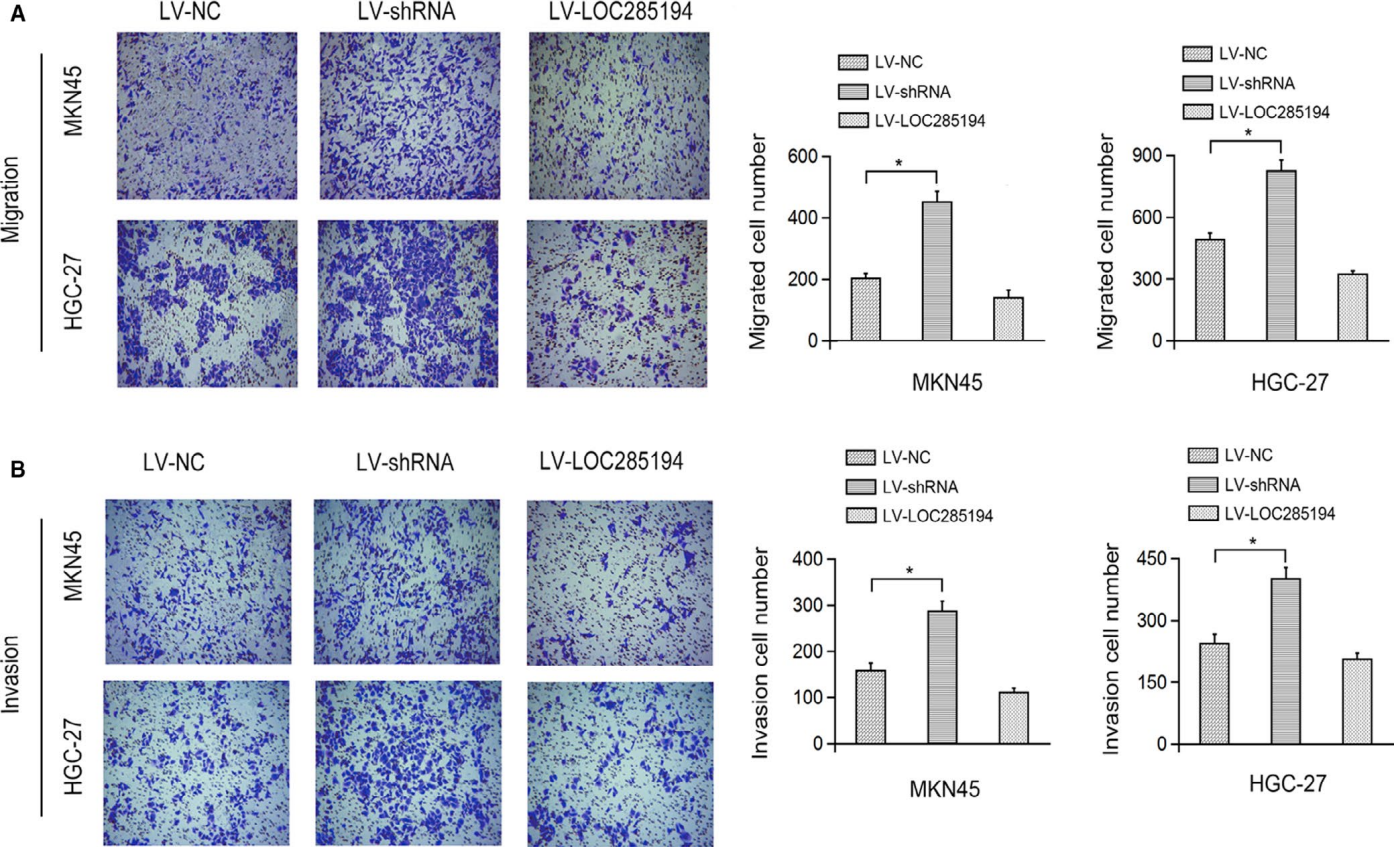
Effects of LOC285194 on GC cell migration and invasion. Effects of LOC285194 on the cell migration (A) and invasion (B) of MKN45 and HGC‐27 cells detected by Transwell assay. *P < .05

Next, EDU and CCK8 assays were conducted to investigate whether LOC285194 affected the cell proliferation of GC cells. LOC285194 was significantly suppressed by LV‐shRNA, whereas significantly promoted by LV‐LOC285194 treatment in MKN45 as well as HGC‐27 cells (Figure 2A). Moreover, CCK8 assay showed that LOC285194 overexpression suppressed GC cell proliferation, meanwhile LOC285194 knockdown promoted cell proliferation of GC cells (Figure 2B). Furthermore, EDU detection revealed that the proliferative rate was markedly repressed by LOC285194 overexpression, but was enhanced by silencing LOC285194 (Figure 2C‐D) in MKN45 and HGC‐27 cell lines. Additionally, the colony formation test revealed that the cell formation ability was significantly promoted by LV‐shRNA, while it was significantly attenuated by LV‐LOC285194 (Figure 3A). In conclusion, the results strongly demonstrated that LOC285194 dramatically restrained GC cell proliferation. Besides, we observed that LV‐shRNA significantly inhibited the apoptosis of MKN45 and HGC‐27 cells while it was induced by LV‐LOC285194 (Figure 3B). Flow cytometric analysis also demonstrated that cell cycle arrest was dramatically attenuated by LV‐LOC285194 (Figure 3C). Transwell assay showed that cell migration and invasion abilities were significantly promoted by LV‐shRNA, but attenuated by LV‐LOC285194 (Figure 4). Taken together, the above results suggested that LOC285194 promoted cell apoptosis and inhibited the cell migration, proliferation, and invasion in GC cells.

### 3.3 LOC285194 downregulated WNT/β‐catenin signaling transduction in GC cells

**Figure 5:**
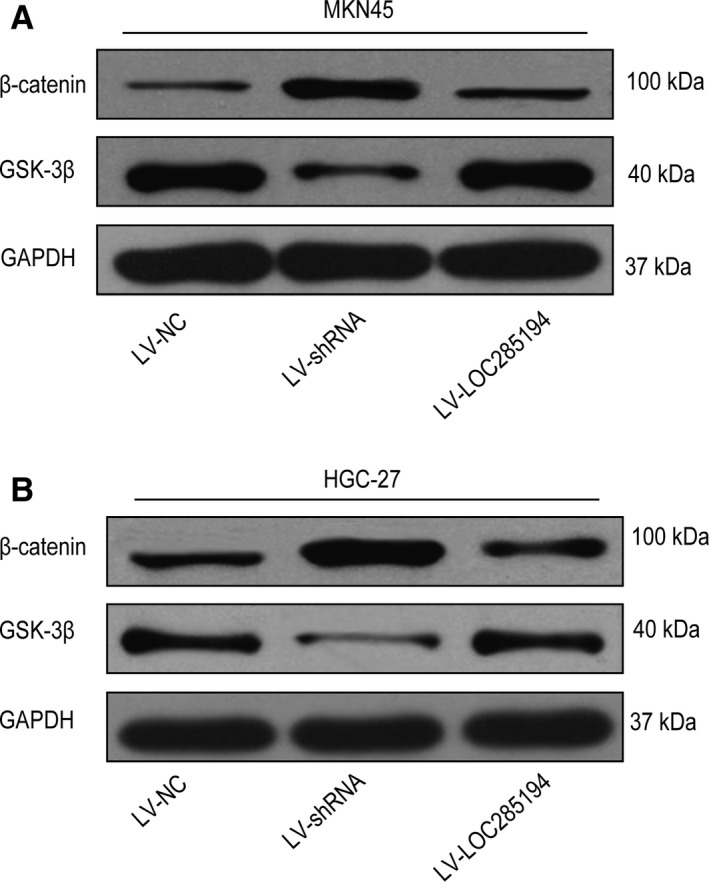
Effects of LOC285194 on the Wnt signaling pathway in GC cells. Protein expressions of β‐Catenin and GSK‐3β in MKN45 cells (A) and HGC‐27 cells (B) detected by western blotting

To further uncover the critical role of LOC285194 in suppressing Wnt signaling pathway in GC progression, the mRNA and protein levels of key elements in Wnt signaling pathway were detected after silencing of LOC285194. Western blotting analysis showed LOC285194 knockdown upregulated the β‐catenin level and downregulated the GSK‐3 β protein level in MKN45 and HGC‐27 cells (Figure 5A‐B). On the contrary, LOC285194 overexpression inactivated Wnt signaling transduction. Therefore, LOC285194 potentially mediated Wnt signaling transduction in GC cells.

### 3.4 LOC285194 inhibited GC progression through WNT signaling transduction in vivo

**Figure 6:**
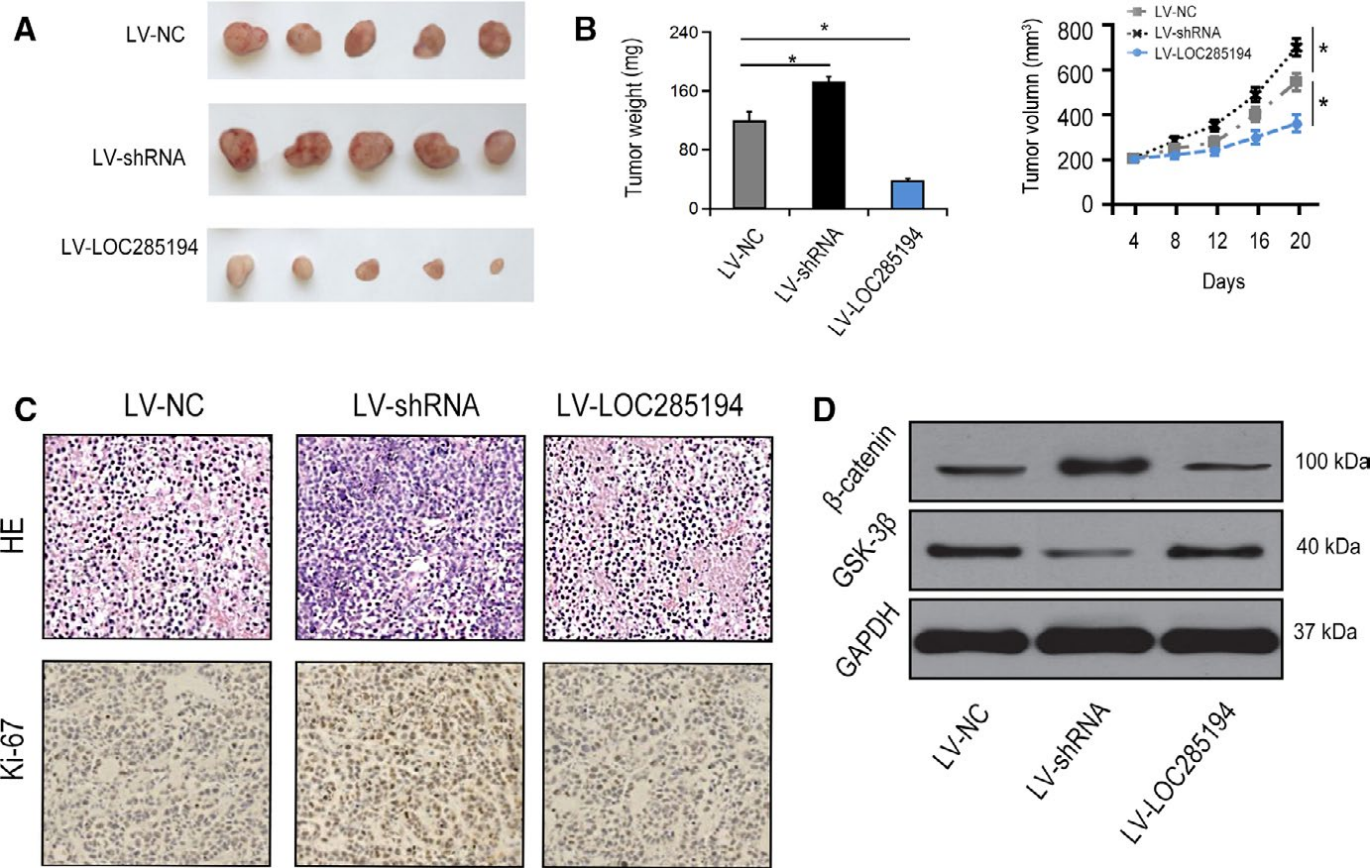
LOC285194 modulated GC progression by regulating Wnt signaling in vivo. BALB/c nude mice were injected with HGC‐27 cells infected with LV‐NC or LV‐shRNA. A‐B, Solid tumors were picked from mouse subcutaneous xenograft tissues. C, Ki‐67 expression in xenograft tumor tissues detected by H&E staining and IHC staining. D, Protein expressions of β‐Catenin and GSK‐3β in xenograft tumor tissues. *P < .05

Next, we confirmed whether LOC285194 regulated GC development in vivo with the xenotransplantation model of HGC‐27 cells in nude mice. Mice were inoculated with HGC‐27 cells in LV‐NC group, LV‐shRNA group and LV‐LOC285194 group. After that, we dissected the tumors, which were shown in Figure 6A. As to the phenotype of tumor, LV‐LOC285194 suppressed the growth of tumors, while LOC285194 knockdown increased the weights and volumes of tumors (Figure 6B). Besides, HE staining and immunohistochemical staining revealed that Ki‐67 was dramatically suppressed in LV‐LOC285194 group by comparison with LV‐shRNA group. Furthermore, LOC285194 significantly inhibited Wnt signaling pathways (Figure 6C‐D). Together, the data suggested that LOC285194 overexpression inhibited GC progression in vivo through modulating Wnt signaling pathway.

## 4 DISCUSSIONS

Surging numbers of lncRNAs are participated in the pathological progression of GC progression. For example, the binding of EZH2 to lncRNA (AGAP2‐AS1) in GC promotes the invasion of GC cells via binding enhancers of zeste homolog 2 (EZH2) while suppressing E‐cadherin.^12^ HNRNPKP2 contributes to the progression of GC, which serves as an indicator of poor prognosis.^13^ Additionally, the elevated AK023391 is related to poor prognosis of GC.^14^ Here in our investigation, the biological role of LOC285194 in GC was elucidated. Importantly, LOC285194 serves as a carcinoma inhibitor by targeting p53 through KRAS/BRAF/SMEK pathway in nonsmall cell carcinoma of lung.^15^ In esophageal squamous cell carcinoma, LOC285194 also participates in chemoradiotherapy resistance.^16^ In our investigation, we determined that LOC285194 was notably downregulated within GC cells. Furthermore, silencing of LOC285194 significantly induced GC cell progression, while LOC285194 overexpression significantly inhibited the progression of GC. In the future investigation, specific microRNA would be of great interest to be further explored to uncover the underlying role of LOC285194 in GC progression.

In our investigation, we also observed a significant attenuation of LOC285194 and a strong activation of Wnt signaling pathway in GC cells. Silencing of LOC285194 promoted GC cell colony formation, proliferation, invasion and migration, and inhibited the apoptosis of cells, whereas LOC285194 overexpression inhibited GC development. Mechanistically, LOC28519 suppressed Wnt signaling pathway via downregulating β‐catenin expression and increasing the expression of GSK‐3β. Furthermore, we carried out in vivo trials to determine the function of LOC285194 and Wnt signaling transduction regarding GC progression.

Wnt signal transduction is highly conservative, which controls various biological processes in the progression of multiple diseases.^17^, ^18^ Furthermore, Wnt signaling transduction is an essential cascade and is closely related to cancer development. For instance, lncRNA TCF7 promotes hepatocellular carcinoma stem cells via activating Wnt signaling transduction.^19^ Besides, lncRNA UCA1 suppresses the growth of esophageal squamous cell carcinoma (ESCC) via modulating Wnt signaling pathway, and CTD903 overexpression suppresses colorectal cancer progression via blocking Wnt pathway.^20^, ^21^ In cervical cancer, the upregulation of NNT‐AS1 enhances cell progression via Wnt signaling transduction.^22^ Meanwhile, in glioma cells lncRNA BLACAT1 facilitates GC development via activating Wnt signaling pathway, and CCAT‐1 promotes cell proliferation and suppresses the apoptosis of cells through Wnt/β‐catenin signaling transduction.^23^ In our present investigation, Wnt signaling activation has been demonstrated to promote GC metastasis, and LOC285194 suppresses GC progression by blocking Wnt signaling pathway activation. Since several signaling pathways might be associated with GC, it is of great importance to pay closer attention to other potential signaling pathways that may be related to GC progress in our future investigations.

Altogether, LOC285194 serves as an inhibitor of GC development and progression via modulating Wnt signaling transduction. It is worthy to note that the downregulation of LOC285194 facilitates GC progress via activating Wnt signaling transduction.

## AUTHOR'S CONTRIBUTION

Conception and intellectual input: CJ; design and performance of experimentation: ZBZ and WQ; manuscript drafting: WQ. Statistical analyses and data interpretation: HJL and XY; All authors read and approved the final manuscript.

## Data Availability

The authors declare that the data supporting the findings of this study are available within the article.
