# Supplementary material for: LncRNA LOC285194 modulates gastric carcinoma progression through activating Wnt/β‐catenin signaling pathway
Source: Cancer Med. 2020 Jan 28;9(6):2181–9. doi: 10.1002/cam4.2844 (PMC7064030; doi:10.1002/cam4.2844)
Supplement: Supplementary file 1 [file CAM4-9-2181-s001.doc]

**Supplementary Table 1** Correlation between LOC285194 expression and clinicopathological characteristics of GC patients (n=72).

| **Characteristics** |  | LOC285194 | | P value |
| --- | --- | --- | --- | --- |
|  |  | High | Low |  |
| **Age (years)** | <50 | 13 | 20 | 0.394 |
|  | ≥50 | 23 | 16 |  |
| **Gender** | Male | 18 | 22 | 0.295 |
|  | Female | 18 | 14 |  |
| **Location** | Distal | 14 | 17 | 0.667 |
|  | Middle | 13 | 11 |  |
|  | Proximal | 9 | 8 |  |
| **Tumor size (cm)** | <5 | 25 | 12 | 0.028^*^ |
|  | ≥5 | 11 | 24 |  |
| **Histologic type** | Well | 3 | 4 | 0.036^*^ |
|  | Moderately | 14 | 9 |  |
|  | Poorly | 9 | 17 |  |
|  | Undifferentiated | 10 | 6 |  |
| **Invasion depth** | T1 | 11 | 5 | 0.004^*^ |
|  | T2 | 14 | 7 |  |
|  | T3 | 4 | 13 |  |
|  | T4 | 4 | 11 |  |
| **Lymphatic metastasis** | NO | 24 | 10 | 0.008^*^ |
|  | YES | 12 | 26 |  |
| **Distant metastasis** | NO | 36 | 33 | 0.123 |
|  | YES | 0 | 3 |  |
| **TNM stage** | I | 6 | 2 | 0.003^*^ |
|  | II | 15 | 7 |  |
|  | III | 14 | 23 |  |
|  | IV | 1 | 4 |  |
